# ﻿A new species of shrew moles, genus *Uropsilus* Milne-Edwards, 1871 (Mammalia, Eulipotyphla, Talpidae), from the Wuyi Mountains, Jiangxi Province, eastern China

**DOI:** 10.3897/zookeys.1186.111592

**Published:** 2023-12-07

**Authors:** Xueyang Ren, Yifan Xu, Yixian Li, Hongfeng Yao, Yi Fang, Laxman Khanal, Lin Cheng, Wei Zeng, Xuelong Jiang, Zhongzheng Chen

**Affiliations:** 1 Collaborative Innovation Center of Recovery and Reconstruction of Degraded Ecosystem in Wanjiang Basin Co-founded by Anhui Province and Ministry of Education, School of Ecology and Environment, Anhui Normal University, Wuhu 241002, China Anhui Normal University Wuhu China; 2 State Key Laboratory of Genetic Resources and Evolution & Yunnan Key Laboratory of Biodiversity and Ecological Security of Gaoligong Mountain, Kunming Institute of Zoology, Chinese Academy of Sciences, Kunming, Yunnan 650204, China Kunming Institute of Zoology, Chinese Academy of Sciences Kunming China; 3 Jiangxi Wuyi Mountain National Nature Reserve, Shangrao 334000, China Jiangxi Wuyi Mountain National Nature Reserve Shangrao China; 4 Central Department of Zoology, Institute of Science and Technology, Tribhuvan University, Kathmandu 44618, Nepal Tribhuvan University Kathmandu Nepal; 5 Southwest Survey and Planning Institute of National Forestry and Grassland Administration, Kunming 650216, China Southwest Survey and Planning Institute of National Forestry and Grassland Administration Kunming China

**Keywords:** Mount Huanggang, small mammals, taxonomy, Uropsilinae

## Abstract

Asian shrew moles, genus *Uropsilus*, are the most primitive members of family Talpidae. They are distributed mainly in southwestern China and adjacent Bhutan, Myanmar, and Vietnam. In June 2022, we collected five specimens of *Uropsilus* from Mount Huanggang, Jiangxi Province, eastern China, which is the highest peak of the Wuyi Mountains. We sequenced two mitochondrial (*CYT B* and *12S rRNA*) and three nuclear (*PLCB4*, *RAG1*, and *RAG2*) genes to estimate the phylogenetic relationship of the five shrew moles. We also compared their morphology with recognized species within the genus. Our results show that these specimens collected from Mount Huanggang differ from all named species in *Uropsilus*. We formally describe the species here as *Uropsilushuanggangensis***sp. nov.** Morphologically, the new species is distinguishable from the other *Uropsilus* species by the combination of dark chocolate-brown pelage, long snout, enlarged first upper incisor, similarly sized lacrimal and infraorbital foramens, and the curved and sickle-like coronoid process. The genetic distances of the cytochrome b (*CYT B*) gene between *U.huanggangensis* and other recognized *Uropsilus* species ranged between 9.3% and 16.4%. The new species is geographically distant from other species in the genus and is the easternmost record of the *Uropsilus*. The divergence time of *U.huanggangensis* was estimated to be the late Pliocene (1.92 Ma, 95% CI = 0.88–2.99).

## ﻿Introduction

The shrew moles of the genus *Uropsilus* Milne-Edwards, 1871 are the sole living genus in the subfamily Uropsilinae in Talpidae (Mckenna et al. 1997; Hutterer 2005). These insectivores primarily inhabit the mountains of southwestern China, as well as adjacent areas in Bhutan and northeastern Myanmar, where they inhabit montane forests at 1,400–3,600 m elevation (IUCN 2015; Hoffmann and Lunde 2008). Although the fossil record is sparse, the age of the associated fossils and the timing of the molecular evolution of mammals suggest that the subfamily Uropsilinae would have flourished and spread widely across Eurasia before the Late Miocene (Meredith et al. 2011). In contrast to other moles that have developed adaptive features such as broad front claws and reduced external ears, shrew moles exhibit shrew-like characteristics, including slender front claws, exposed external ears, and long tails almost equal in length to their bodies. All these morphological characteristics suggest that they have retained the terrestrial habits of primitive moles (Allen 1938). Phylogenetic relationships constructed using morphological methods (Motokawa 2004; Sánchez‐Villagra et al. 2006) and molecular phylogenetic methods (Douady and Douzery 2003; Shinohara et al. 2003) consistently support that *Uropsilus* forms the basal branch in the phylogenetic tree of the family Talpidae.

The genus *Uropsilus* was first described by Milne-Edwards (1871) based on the specimens from Muping (= Baoxing) in Sichuan, China. The type species of the *Uropsilus* is *U.soricipes* Milne-Edwards, 1871, which has a dental formula of: I 2/1, C 1/1, P 3/3, M 3/3 = 34. Thomas (1912) described two new species belonging to two new genera: *Rhynchonaxandersoni* Thomas, 1911 from Mount Omi-san (= Mount Emei), Sichuan, with the dental formula: I 2/2, C 1/1, P 4/3, M 3/3 = 38; and *Nasillusgracilis* Thomas, 1911 from Chin-fu-san (= Jinfo shan), Chongqing, with the dental formula: I 2/1, C 1/1, P 4/4, M 3/3 = 38. Later, Thomas (1922) described *N.investigator* Thomas, 1922, based on larger specimens collected in the Kia-kiang-Salween of Yunnan compared to *N.gracilis*. Additionally, Allen (1923) described two new subspecies of *R.andersoni*: *R.andersoniatronates* Allen, 1923 from Salween drainage, Yunnan; and *R.andersoninivatus* Allen, 1923 from Lijiang, Yunnan, China. However, the classification of the shrew moles into three genera had been widely disputed. Osgood (1937) considered two genera (*Uropsilus* Milne-Edwards, 1871 and *Nasillus* Thomas, 1911) in the Uropsilinae and merged the genus *Rhynchonax* Thomas, 1911 into the genus *Uropsilus*. Ellerman and Morrison-Scott (1951) assigned all genera to *Uropsilus* and placed the named species in five subspecies of *U.soricipes*: *U.s.soricipes*, *U.s.gracilis*, *U.s.andersoni* (including *atronates*), *U.s.investigator* and *U.s.nivatus*. The proposition of one genus is recognized by most scholars (Cranbrook 1960–1961; Corbet and Hill 1980; Honacki et al. 1982; Hutterer 2005). Hoffmann (1984) conducted a systematic study of this group and recognized three species: *U.soricipes*, *U.gracilis*, and *U.andersoni* under the single genus *Uropsilus*. However, Wang and Yang (1989) believed that *investigator* and *gracilis* were distributed in the same domain, and there was no intermediate transition type, so the *U.s.investigator* should be an independent species. Since then, the view that there are four species of *Uropsilus* has been widely accepted (Hoffmann and Lunde 2008).

Recently, Liu et al. (2013) described *U.aequodonenia*Liu et al., 2013 from Puge County, Sichuan, China, which has a dental formula of I 2/2, C1/1, P3 /3, and M 3 /3 = 36. Wan et al. (2013) suggested that *U.nivatus* and *U.atronates* are valid species based on molecular data. Furthermore, they identified seven recognized species and five putative species. Wan (2015) described six new species, but these designations were not recognized because they did not follow the International Code of Zoological Nomenclature (ICZN 2012). Wan et al. (2018) generated gene trees using additional specimens, which phylogenetic analyses revealed that species of *Uropsilus* could be sorted into three distinct lineages. One lineage includes *U.investigator* from western Yunnan and acts as the basal position of the genus; the second lineage includes *U.aequodonenia*, *U.andersoni*, and *U.nivatus* from Northern Yunnan and western Sichuan; and the third contains *U.soricipes*, *U.gracilis*, *U.atronates*. Additionally, Hu et al. (2021b) used multivariate analyses as well as phylogenetic analyses to describe a new species, *U.dabieshanensis* Hu et al., 2021, from the Dabie Mountains, Anhui Province, eastern China. The phylogenetic results indicate that the lineage of *Uropsilus* has two matrilines. More recently, Bui et al. (2023) described a new species, *U.fansipanensis*, from the northwestern Vietnam. Thus, nine taxa are currently recognized as full species under the genus *Uropsilus*: *U.aequodonenia*, *U.andersoni*, *U.gracilis*, *U.investigator*, *U.soricipes*, *U.atronates*, *U.nivatus*, *U.dabieshanensis*, and *U.fansipanensis*. Of all known species, *U.dabieshanensis* is the only one found in eastern China and is considered to have the easternmost distribution of the genus.

**Figure 1.**
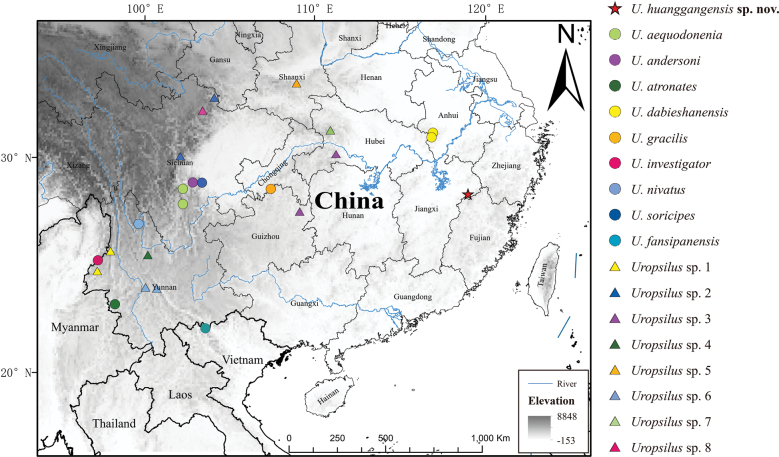
Sampling localities of specimens used in the phylogenetic analysis.

During a biodiversity study in June 2022, five shrew mole specimens were collected from the Wuyi Mountains, Jiangxi Province, eastern China (Fig. 1). Our molecular analysis reveals that the five specimens are genetically distinct from all recognized *Uropsilus* species and potentially represents a new species. In this study, we integrate genetic and morphometric approaches to elucidate the taxonomy and phylogeny of these specimens.

## ﻿Materials and methods

### ﻿Sampling

In June 2022, five *Uropsilus* specimens were collected on Mount Huanggang in Wuyishan National Nature Reserve, Yanshan, Jiangxi Province, eastern China (Fig. 1). Specimens were collected using Sherman and pitfall (plastic buckets 15 cm in diameter and 28 cm in depth) traps. All specimens were euthanized, and muscle or liver tissue was extracted from each and preserved in pure alcohol for subsequent molecular studies. All specimens and tissues were deposited at the Biological Museum of Anhui Normal University (**AHNU**). Animals were handled in compliance with the animal care and use guidelines of the American Society of Mammologists (Sikes et al. 2016), following the guidelines and regulations approved by the internal review board of AHNU (approval no. AHNU-ET2021002), and with the permissions of local government authorities.

### ﻿Phylogenetic analyses

Genomic DNA of the five specimens of *Uropsilus* from Mount Huanggang was extracted from the liver and muscle tissues using a DNA extraction kit (Tiangen DNeasy Blood and Tissue Kit, Beijing, China). Two mitochondrial genes (cytochrome b [*CYT B*], 12S rRNA [*12S*]) and three nuclear genes (phospholipase C beta 4 [*PLCB4*], recombination activating protein 1 [*RAG1*], and recombination activating protein 2 [*RAG2*]) were amplified using the primer pairs outlined in Suppl. material 1. The PCR products were purified and sequenced in both directions using the BigDye Terminator Cycle Kit v. 3.1 (Invitrogen, Waltham, MA, USA) on an ABI 3730xl sequencer (Applied Biosystems, Waltham, MA, USA). The obtained sequences were assembled using SeqMan (DNASTAR, Lasergene v. 7). Corresponding sequences of 38 specimens of nine recognized species and six unrecognized species of *Uropsilus* were downloaded from the GenBank (Suppl. material 2). We downloaded sequences of *Talpaaltaica* and *Sorexaraneus* as out-group taxa following Wan et al (2018). All sequences were then aligned in MEGA v. 11 (Tamura et al. 2021).

The uncorrected *p*-distance of the *CYT B* gene between species was calculated in MEGA v. 11 (Tamura et al. 2021). We used maximum likelihood (ML) and Bayesian inference (BI) methods to conduct phylogenetic analyses of mitochondrial–nuclear genes (mtDNA + nDNA, 4090 bp) concatenated datasets in PhyloSuite (Zhang et al. 2020). The best-fit partitioning scheme and evolutionary models were selected using PartitionFinder v. 2.0 with the greedy algorithm under the Bayesian information criterion (BIC) (Suppl. material 3) (Lanfear et al. 2012).

### ﻿Molecular dating

We used BEAST v. 2.6.6 (Bouckaert et al. 2019) to estimate divergence times based on the Birth-Death model as the tree prior and relaxed lognormal as the clock model prior. Evolutionary models or partition schemes were estimated based on the Bayesian Information Criterion (BIC) in PartitionFinder v. 2.0 (Lanfear et al. 2012). Two fossil calibrations were used following the guide of Wan et al. (2018): (1) the first division of *Uropsilus* at 6.18 Ma (95% HPD: 4.27–8.65 Ma), with a lognormal distribution prior (mean: 6.20, SD: 0.215, offset: 0.02), so the median age was at 6.08 Ma and the 95% CI was 4.27–8.65 Ma; (2) the earliest known *U.soricipes* from the Early Pleistocene 2.0–2.4 Ma, with an exponential distribution prior (offset = 2.0, M = 0.67 [2.0 × 0.333]), so the median age was 2.46 Ma and the 95% CI was 2.03–4.01 Ma. Each analysis was run for 100 million generations, sampling every 10000 generations. The first 10% of the samples were discarded as burn‐in. Convergence was assessed using Tracer v. 1.7 (Rambaut et al. 2018).

### ﻿Morphological measurements and analyses

We examined and measured the specimens of *Uropsilus* in the Kunming Institute of Zoology (KIZ), Chinese Academy of Sciences, and AHNU. A total of 83 specimens were examined and they were assigned to *U.aequodonenia* (*n* = 2), *U.andersoni* (*n* = 6), *U.atronates* (*n* = 25), *U.dabieshanensis* (*n* = 6), *U.gracilis* (*n* = 16), *U.investigator* (*n* = 11), *U.nivatus* (*n* = 7), *U.soricipes* (*n* = 5), and *Uropsilus* sp. nov. (*n* = 5) (Appendix 1).

The body weight (**Wt**) and four external measurements, including head and body length (**HBL**), tail length (**TL**), hindfoot length (**HF**), and ear length (**EL**), were taken from specimen labels or field notes. Twenty-one craniodental measurements were taken with digital calipers to the nearest 0.01 mm, following Yang et al. (2005, 2007). All the craniodental measurements were taken by a single observer. The following measurements were taken:

**PL** Profile length;

**HB** Height of braincase;

**GNB** Greatest neurocranium breadth Cranial breadth;

**BS** Basion-Staphylion;

**GBSn** Greatest breadth of snout;

**BBP^1^**–**P^2^** Maxillary sides P^1^-P^2^ Interdental external width;

**MPL** Palatal length;

**APB** Anterior palatal breadth;

**LUTR** Length of upper tooth row;

**PPB** Posterior palatal breadth;

**P^4^**–**M^3^** Distance from the upper fourth premolar to the upper third molar;

**Id–Gol** From infradentale to gonion laterale;

**M^1^**–**M^3^** Upper molar row length;

**HVR** Oral height of the vertical ramus;

**GBUM** Great breadth of upper molars;

**Coh**–**M_3_** From the highest point of the Condyle process to the upper third molar;

**ML** Mandible length;

**GBLM** Greatest breadth of lower molars;

**Id–Coh** From infradentale to the hight point of the condyle process;

**LBO** least breadth between orbits;

**LBTR** Length of below tooth row.

We compared morphology of the new species with other species of *Uropsilus*. Comparative morphological characters of these other species were obtained from Thomas (1912, 1922), Allen et al. (1923), Hu et al. (2021b), and Bui et al. (2023), and we followed these authors’ terminologies in our morphological description of the new species. Meanwhile, to better distinguish between the different species, we compared the ratio of GNB to PL, as well as the ratio of TL to HBL.

Overall similarities of skulls were assessed first through principal component analyses (PCA) based on the 21 log_10_-transformed craniodental variables. Groups of individuals sharing a comparable morphology were then discriminated through discriminant analysis (DA). To make the results more concise, we limited the PCA and DA analyses to the six taxa with the same dental formula, including: *U.atronates*, *U.dabieshanensis*, *U.gracilis*, *U.investigator*, *U.nivatus*, and the new species. The PCA and DA were conducted in SPSS v. 22.0 (SPSS Inc., USA). Furthermore, independent sample *t*-tests were conducted to test the variances of measurements highly correlated with PC1 and PC2 (i.e. LUTR, LBTR, GBSn, and GBUM; loading > 0.8) between the new species and the other species.

## ﻿Results

### ﻿Phylogenetic analyses

We obtained 4090-bp-long sequences for each voucher specimen, including 2002-bp mitochondrial [*CYT B* (1140 bp) and *12S* (862 bp)] and 2088-bp nuclear [*PLCB4* (330 bp), *RAG1* (1008 bp), and *RAG2* (750 bp)] sequences. All the new sequences were deposited in the GenBank [OQ730193–OQ730207, OQ725651–OQ725655, OR161365–OR161369, Suppl. material 2]. The uncorrected *p*-distance of *CYT B* reveals a high genetic divergence between the new species and all other nominal *Uropsilus* species, ranging from 9.3% (with *U.gracilis*) to 16.4% (with *U.investigator*) (Table 1).

**Table 1.** The uncorrected *p*-distances between *Uropsilus* species/cryptic species based on the *CYT B* gene.

	Species	1	2	3	4	5	6	7	8	9	10	11	12	13	14	15	16	17
1	*U.huanggangensis* **sp. nov.**																	
2	*U.gracilis*	0.093																
3	*U.soricipes*	0.094	0.084															
4	*U.fansipanensis*	0.094	0.092	0.091														
5	*U.atronates*	0.100	0.097	0.095	0.095													
6	*U.dabieshanensis*	0.104	0.097	0.105	0.108	0.125												
7	*U.nivatus*	0.133	0.113	0.126	0.130	0.133	0.139											
8	*U.aequodonenia*	0.138	0.130	0.122	0.143	0.142	0.147	0.103										
9	*U.andersoni*	0.139	0.134	0.132	0.138	0.144	0.141	0.099	0.079									
10	*U.investigator*	0.164	0.164	0.155	0.180	0.175	0.158	0.163	0.167	0.165								
11	*U.* sp. 1	0.161	0.157	0.154	0.172	0.160	0.155	0.157	0.156	0.162	0.097							
12	*U.* sp. 2	0.134	0.130	0.135	0.133	0.145	0.128	0.133	0.137	0.129	0.160	0.158						
13	*U.* sp. 3	0.100	0.108	0.104	0.108	0.104	0.114	0.126	0.142	0.139	0.158	0.160	0.135					
14	*U.* sp. 4	0.091	0.077	0.088	0.083	0.097	0.118	0.116	0.135	0.133	0.164	0.165	0.132	0.089				
15	*U.* sp. 5	0.097	0.086	0.039	0.094	0.101	0.097	0.127	0.128	0.135	0.154	0.150	0.135	0.101	0.090			
16	*U.* sp. 6	0.094	0.091	0.091	0.038	0.093	0.110	0.132	0.144	0.141	0.172	0.172	0.130	0.100	0.082	0.090		
17	*U.* sp. 7	0.088	0.087	0.084	0.088	0.099	0.099	0.127	0.133	0.132	0.157	0.157	0.131	0.096	0.085	0.079	0.095	
18	*U.* sp. 8	0.139	0.120	0.133	0.137	0.136	0.138	0.121	0.122	0.127	0.169	0.156	0.087	0.139	0.137	0.137	0.139	0.130

**Figure 2.**
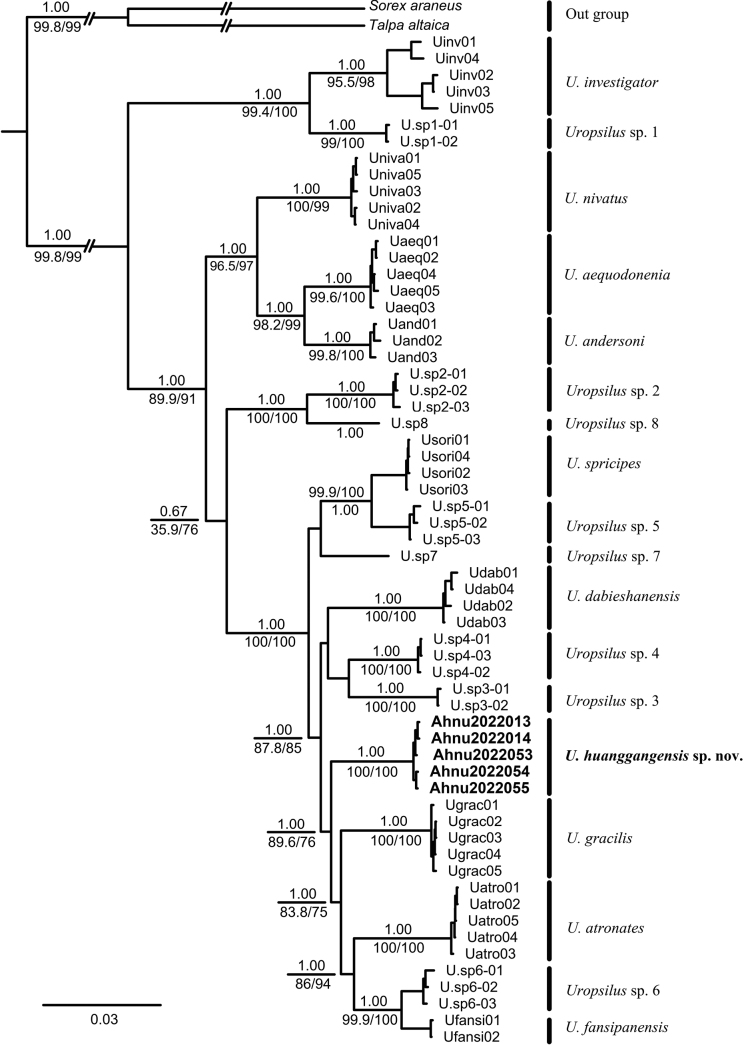
Molecular phylogenetic tree of *Uropsilus* based on mitochondrial–nuclear concatenated data and analyzed using maximum likelihood and Bayesian inference analyses. Numbers above branches refer to Bayesian posterior probabilities (PP). Numbers below branches indicate SH-like approximate likelihood ratio test supports (SH-aLRT)/ultrafast bootstrap supports (UFBoot). Scale bars represent substitutions per site.

**Figure 3.**
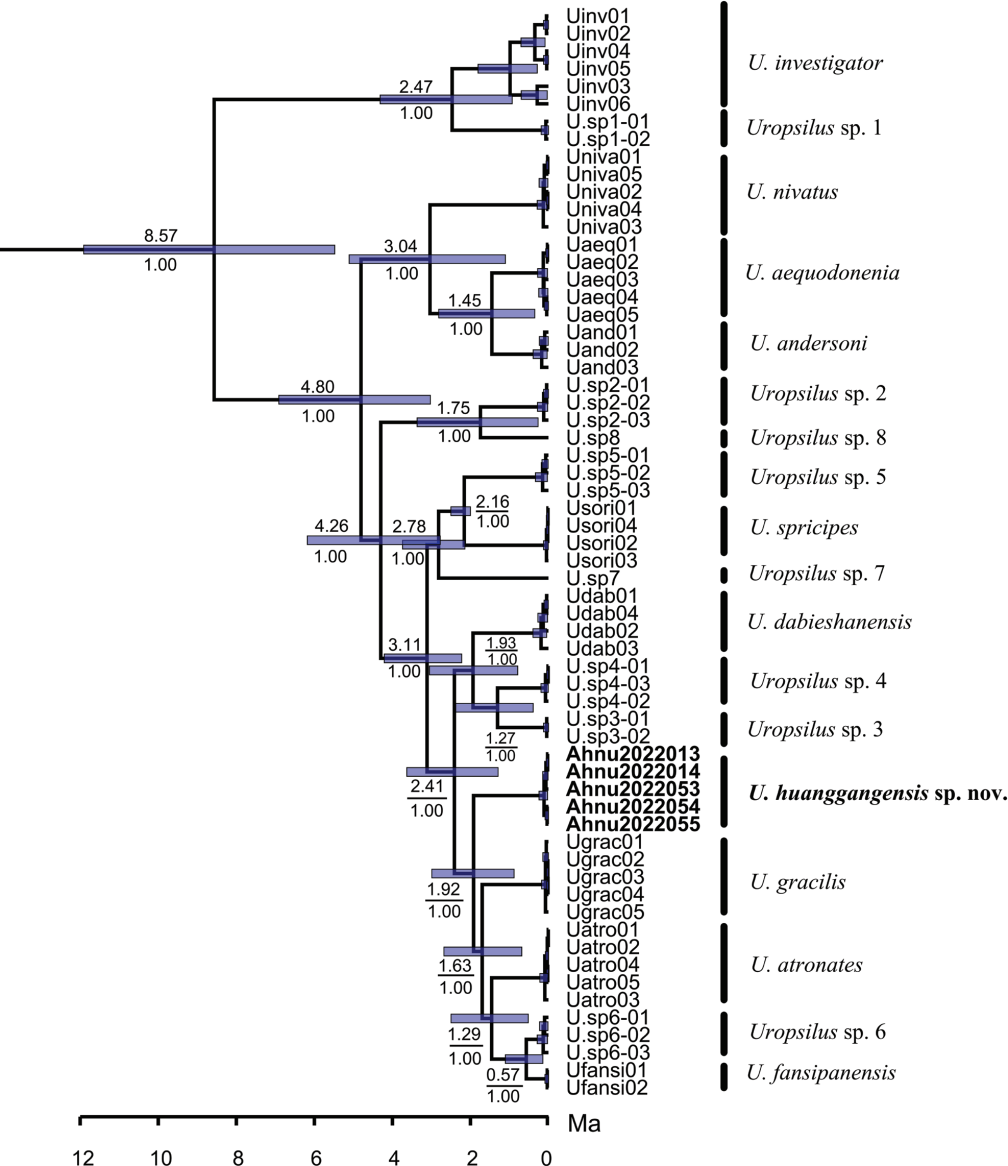
Divergence times estimated using BEAST based on mitochondrial–nuclear concatenated data. Node numbers refer to divergence time in million years (Ma) and Bayesian posterior probabilities (PP).

The ML and BI trees recovered similar topologies (Fig. 2). In all phylogenetic trees, sequences of the new species from Mount Huanggang formed a monophyletic clade with high support (SH-aLRT = 100, Utboot = 100, and PP = 1.00). The new species has a sister relationship with the clade that is comprised of *U.atronates*, *Uropsilus* sp. 6, *U.fansipanensis*, and *U.gracilis*; this clade is strongly supported in the BI tree (PP = 1.0), but this relationship only has moderate support in the ML tree (SH-aLRT = 89.6, Utboot = 76). BEAST divergence analyses show that the divergence of the new species from the others was estimated to be at the early Pleistocene (1.92 Ma, 95% CI = 0.88–2.99) (Fig. 3).

### ﻿Morphological analyses

**Figure 4.**
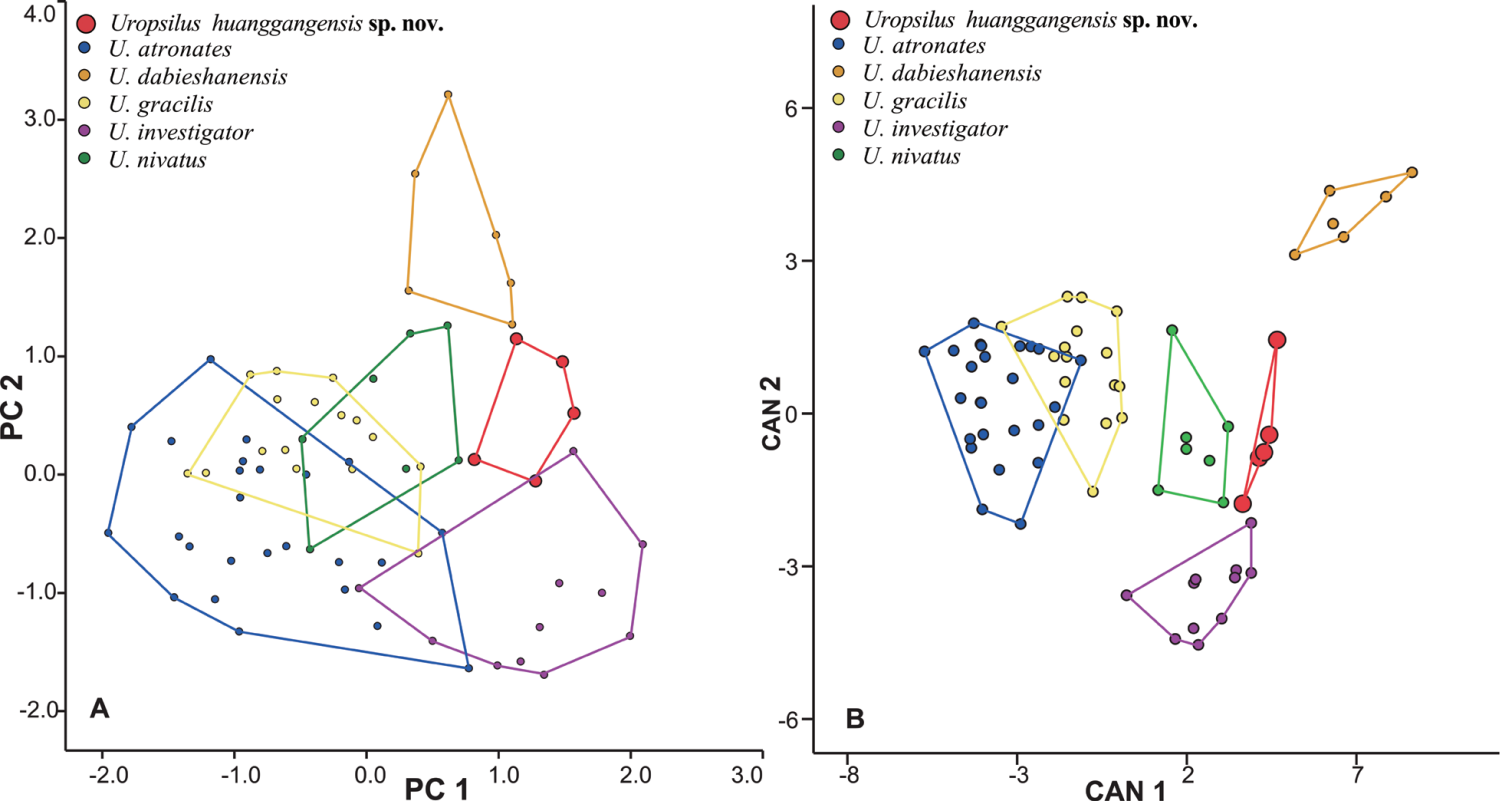
Results of **A** principal component analysis (PCA) **B** discriminant function analysis (DA) for partial species with the same dental formula within the genus *Uropsilus*.

All external and skull measurements are given in Table 2. The PCA, which was based on 21 craniodental measurements, produced two axes with eigenvalues exceeding 2.0, which explained 57.24% and 10.53% of the variance (67.77% total) (Table 3). The first principal component (PC1) is positively correlated with LUTR and LBTR (loading > 0.80), indicating it mainly represents tooth row length. The second principal component (PC2) has high positive loadings on GBSn and GBUM (loading > 0.85). The independent-sample *t*-tests further show significant differences of at least two of the four measurements (i.e. LUTR, LBTR, GBSn, and GBUM) between the new species and *U.atronates*, *U.dabieshanensis*, *U.gracilis*, *U.investigator*, *U.nivatus* (*p* < 0.05; Suppl. material 4). A plot of PC1 and PC2 (Fig. 4) shows *Uropsilus* sp., *U.investigator*, and *U.dabieshanensis* occupy the positive region of PC1, indicating that these three species have longer tooth rows than *U.atronates*, *U.nivatus*, and *U.gracilis*. The new species plots on the near-origin area of PC2, while *U.investigator* occupies the negative regions and *U.dabieshanensis* occupies the positive region, suggesting that the snout and upper molars of the new species are relatively narrower than *U.dabieshanensis* but broader than those in *U.investigator*. The DA shows that 98.60% of the species are correctly classified based on the 21 craniodental measurements, with only one specimen labeled as *U.investigator* misclassified as *U.nivatus*. The first two canonical axes (CAN 1–2) explains 63.30% and 19.40% of the total variation, respectively (Table 3). In CAN 1 and CAN 2 plots (Fig. 4), specimens of the new species are separate from the others and remain close to *U.nivatus* and *U.investigator*.

**Table 2.** External and skull measurements (mm) used in morphometric analyses of the genus *Uropsilus*, including mean values, standard deviations, range, sample size and the ratio of partial measurements.

	*U.huanggangensis* sp. nov.	*U.dabieshanensis*	*U.gracilis*	*U.atronates*	*U.investigator*	*U.nivatus*	*U.aequodonenia*	*U.andersoni*	*U.soricipes*	*U.fansipanensis*
**W**	8.80 ± 0.38	9.28 ± 1.25	8.84 ± 0.95	6.93 ± 0.92	7.25 ± 0.88	8.00 ± 1.38	8	9.04 ± 0.57	9.52 ± 1.34	8.0; 8.0
	8.17–9.19; 5	7.63–10.52; 6	7.30–10.20; 14	5.50–8.80; 15	5.90–8.40; 10	6.40–10.90; 7		8.20–9.80; 5	7.20–11.70; 10	
**HBL**	72.40 ± 1.34	72.67 ± 1.89	69.86 ± 4.15	65.27 ± 2.76	71.65 ± 4.30	69.71 ± 2.14	75	74.33 ± 3.01	73.36 ± 2.69	77.5; 74.00
	71.00–74.00; 5	69.50–75.00; 6	63.00–77.00; 14	61.00–69.00; 15	66.00–79.00; 10	67.00–73.00; 7		70.00–79.00; 6	70.00–79.00; 11	
**TL**	62.60 ± 3.21	55.50 ± 3.35	63.57 ± 6.11	59.73 ± 4.45	65.40 ± 4.30	68.86 ± 3.98	70	65.17 ± 10.68	63.18 ± 8.21	62.50; 61.00
	57.00–65.00; 5	52.50–61.50; 6	53.00–75.00; 14	52.00–68.00; 15	60.00–71.00; 10	61.00–73.00; 7		46.00–73.00; 6	46.00–73.00; 11	
**HF**	13.10 ± 0.22	11.75 ± 1.21	12.96 ± 0.97	12.80 ± 0.77	13.20 ± 1.40	14.14 ± 1.68	14	14.75 ± 0.76	14.05 ± 0.99	13.41; 13.57
	13.00–13.50; 5	10.50–13.00; 6	10.00–14.00; 14	11.00–14.50; 15	11.00–15.00; 10	11.00–16.00; 7		14.00–16.00; 6	13.00–16.00; 11	
**EL**	9.20 ± 0.45	8.33 ± 0.41	9.39 ± 1.1	8.07 ± 0.86	7.95 ± 0.98	9.50 ± 0.84	11	9.67 ± 0.58	9.38 ± 0.74	8.51; 8.52
	9.00–10.00; 5	8.00–9.00; 6	7.00–11.00; 14	7.00–9.50; 15	6.50–9.50; 10	8.00–10.00; 6		9.00–10.00; 3	8.00–10.00; 8	
**PL**	21.44 ± 0.47	21.04 ± 0.45	20.89 ± 0.41	20.12 ± 0.43	21.19 ± 0.71	20.78 ± 0.29	21.91	22.06 ± 0.33	21.05 ± 0.23	20.4; 20.69
	21.00–22.18; 5	20.32–21.50; 6	19.93–21.81; 16	18.99–20.79; 25	19.97–22.14; 11	20.39–21.12; 7	21.91; 1	21.59–22.34; 3	20.63–21.3; 5	
**GNB**	11.10 ± 0.17	11.52 ± 0.29	11.27 ± 0.20	10.91 ± 0.23	11.10 ± 0.34	11.31 ± 0.25	11.90 ± 0.19	11.66 ± 0.18	11.36 ± 0.26	10.96; 11.07
	10.95–11.39; 5	11.00–11.86; 6	10.88–11.63; 16	10.62–11.31; 25	10.45–11.64; 11	10.98–11.66; 7	11.71; 12.09; 2	11.49–11.91; 3	10.94–11.73; 5	
**GBSn**	7.62 ± 0.11	8.10 ± 0.34	7.57 ± 0.18	7.27 ± 0.21	7.27 ± 0.28	7.82 ± 0.24	8.12 ± 0.02	7.87 ± 0.21	7.70 ± 0.14	7.39; 7.62
	7.47–7.77; 5	7.65–8.51; 6	7.23–7.89; 16	6.84–7.72; 25	6.92–7.81; 11	7.51–8.15; 7	8.10; 8.14; 2	7.69–8.26; 6	7.52–7.92; 5	
**MPL**	9.85 ± 0.29	9.79 ± 0.18	9.39 ± 0.17	8.90 ± 0.24	9.72 ± 0.38	9.59 ± 0.22	10.11 ± 0.13	10.00 ± 0.15	9.72 ± 0.08	9.62; 9.75
	9.50–10.21; 5	9.46–9.96; 6	9.18–9.68; 16	8.49–9.33; 25	9.19–10.25; 11	9.31–9.90; 7	9.98; 10.24; 2	9.75–10.22; 5	9.65–9.85; 5	
**LUTR**	9.47 ± 0.24	9.46 ± 0.27	9.02 ± 0.17	8.65 ± 0.26	9.38 ± 0.34	9.17 ± 0.20	9.61 ± 0.10	9.55 ± 0.15	9.24 ± 0.16	9.03; 9.07
	9.12–9.76; 5	9.03–9.68; 6	8.65–9.25; 16	8.23–9.32; 25	8.96–9.93; 11	8.95–9.40; 7	9.51; 9.7; 2	9.24–9.68; 6	9.04–9.49; 5	
**P^4^-M^3^**	5.51 ± 0.14	5.65 ± 0.24	5.33 ± 0.12	5.13 ± 0.19	5.36 ± 0.21	5.37 ± 0.16	5.78 ± 0.07	5.75 ± 0.15	5.58 ± 0.20	
	5.35–5.67; 5	5.27–6.03; 6	5.05–5.55; 16	4.79–5.64; 25	5.04–5.64; 11	5.11–5.55; 7	5.73; 5.86; 2	5.53–5.98; 6	5.26–5.86; 5	
**M^1^-M^3^**	4.53 ± 0.10	4.62 ± 0.21	4.36 ± 0.13	4.17 ± 0.15	4.35 ± 0.17	4.40 ± 0.13	4.51 ± 0.10	4.55 ± 0.2	4.39 ± 0.18	
	4.37–4.62; 5	4.29–4.93; 6	4.11–4.64; 16	3.92–4.62; 25	4.15–4.72; 11	4.22–4.58; 7	4.41; 4.61; 2	4.26–4.92; 6	4.10–4.58; 5	
**GBUM**	1.92 ± 0.06	2.02 ± 0.08	1.84 ± 0.06	1.76 ± 0.07	1.71 ± 0.07	1.83 ± 0.07	1.82 ± 0.03	1.89 ± 0.05	1.82 ± 0.11	
	1.82–1.97; 5	1.91–2.14; 6	1.76–1.95; 16	1.62–1.88; 25	1.63–1.84; 11	1.77–1.94; 7	1.79; 1.84; 2	1.80–1.97; 6	1.72–1.95; 5	
**ML**	13.84 ± 0.26	14.07 ± 0.22	13.53 ± 0.27	12.98 ± 0.36	13.73 ± 0.54	13.67 ± 0.18	14.35 ± 0.08	14.27 ± 0.28	13.81 ± 0.2	13.52; 13.77
	13.46–14.12; 5	13.80–14.41; 6	12.96–13.92; 16	12.36–13.75; 25	12.85–14.34; 11	13.41–13.87; 7	14.27; 14.42; 2	13.94–14.73; 6	13.45–14.02; 5	
**Id-Coh**	12.40 ± 0.35	12.32 ± 0.32	12.55 ± 0.19	12.04 ± 0.33	12.42 ± 0.65	12.21 ± 0.26	12.87 ± 0.41	12.82 ± 0.38	12.44 ± 0.46	
	11.99–12.74; 5	11.82–12.74; 6	12.28–12.94; 16	11.44–12.77; 25	11.34–13.26; 11	11.86–12.60; 7	12.46; 13.28; 2	12.38–13.41; 6	11.8–13.17; 5	
**LBTR**	8.10 ± 0.12	8.10 ± 0.21	7.69 ± 0.21	7.46 ± 0.23	8.04 ± 0.37	7.80 ± 0.19	8.18 ± 0.06	8.01 ± 0.12	7.87 ± 0.22	
	7.93–8.20; 5	7.84–8.42; 6	7.17–8.01; 16	7.04–7.84; 25	7.50–8.54; 11	7.57–8.05; 7	8.12; 8.24; 2	7.84–8.17; 6	7.67–8.22; 5	
**LBO**	5.51 ± 0.12	5.42 ± 0.26	5.33 ± 0.12	5.46 ± 0.18	5.54 ± 0.16	5.6 ± 0.14	5.63 ± 0.07	5.82 ± 0.11	5.47 ± 0.14	5.51; 5.80
	5.32–5.65; 5	5.05–5.77; 6	5.12–5.58; 16	5.12–5.79; 25	5.23–5.86; 11	5.31–5.77; 7	5.56; 5.70; 2	5.64–5.97; 5	5.20–5.61; 5	
**HB**	7.06 ± 0.15	6.84 ± 0.40	7.15 ± 0.26	6.70 ± 0.23	7.16 ± 0.31	7.03 ± 0.15	7.13	7.37 ± 0.28	7.38 ± 0.29	6.90; 6.91
	6.92–7.35; 5	6.08–7.22; 6	6.65–7.65; 16	6.24–7.17; 25	6.62–7.68; 11	6.77–7.17; 7	7.13; 1	7.08–7.75; 3	6.8–7.52; 5	
**BS**	7.57 ± 0.16	7.48 ± 0.24	7.36 ± 0.22	7.27 ± 0.29	7.34 ± 0.19	7.32 ± 0.13	7.57	7.57 ± 0.36	7.50 ± 0.09	
	7.33–7.76; 5	7.16–7.73; 6	6.97–7.96; 16	6.68–7.8; 25	6.88–7.59; 11	7.12–7.54; 7	7.57; 1	7.07–7.93; 3	7.40–7.65; 5	
**BBP^1^-P^2^**	2.96 ± 0.11	3.08 ± 0.17	2.82 ± 0.09	2.68 ± 0.10	2.77 ± 0.09	2.80 ± 0.09	2.99 ± 0.06	2.96 ± 0.05	2.93 ± 0.08	
	2.77–3.05; 5	2.84–3.38; 6	2.67–2.94; 16	2.41–2.88; 25	2.63–2.94; 11	2.63–2.88; 7	2.93; 3.05; 2	2.90–3.03; 6	2.80–3.03; 5	
**APB**	2.58 ± 0.10	2.55 ± 0.13	2.36 ± 0.10	2.33 ± 0.20	2.50 ± 0.10	2.45 ± 0.09	2.51 ± 0.06	2.34 ± 0.13	2.37 ± 0.08	
	2.45–2.72; 5	2.35–2.77; 6	2.26–2.67; 16	2.00–3.03; 25	2.31–2.65; 11	2.30–2.58; 7	2.45; 2.57; 2	2.16–2.47; 6	2.28–2.48; 5	
**PPB**	3.23 ± 0.09	3.29 ± 0.19	3.13 ± 0.10	3.06 ± 0.14	3.13 ± 0.13	3.34 ± 0.18	3.50 ± 0.03	3.27 ± 0.09	3.20 ± 0.04	
	3.10–3.32; 5	2.93–3.54; 6	2.94–3.31; 16	2.68–3.35; 25	2.91–3.34; 11	3.13–3.59; 7	3.47; 3.52; 2	3.18–3.40; 6	3.12–3.24; 5	
**Id-Gol**	12.96 ± 0.05	13.14 ± 0.28	12.06 ± 0.33	11.43 ± 0.43	12.85 ± 0.47	12.69 ± 0.20	13.09 ± 0.10	13.07 ± 0.13	12.83 ± 0.19	
	12.90–13.03; 5	12.66–13.54; 6	11.46–12.68; 16	10.79–12.38; 25	12.18–13.58; 11	12.43–12.98; 7	12.99; 13.18; 2	12.89–13.3; 6	12.61–13.18; 5	
**HVR**	6.31 ± 0.15	6.39 ± 0.31	6.32 ± 0.12	6.00 ± 0.19	6.22 ± 0.29	6.32 ± 0.09	6.73 ± 0.02	6.63 ± 0.15	6.44 ± 0.17	
	6.10–6.57; 5	5.91–6.90; 6	6.09–6.49; 16	5.67–6.49; 25	5.77–6.61; 11	6.19–6.49; 7	6.71; 6.74; 2	6.44–6.84; 6	6.30–6.75; 5	
**Coh-M_3_**	5.79 ± 0.05	5.56 ± 0.29	5.68 ± 0.25	5.36 ± 0.26	5.81 ± 0.32	5.78 ± 0.17	6.45	6.07 ± 0.33	5.90 ± 0.23	
	5.72–5.87; 5	5.18–6.00; 6	5.15–6.11; 16	4.81–5.98; 25	5.25–6.29; 11	5.52–6.00; 7	6.45; 1	5.67–6.55; 6	5.57–6.22; 5	
**GBLM**	1.19 ± 0.05	1.25 ± 0.06	1.14 ± 0.04	1.09 ± 0.05	1.09 ± 0.04	1.14 ± 0.05	1.19 ± 0.03	1.20 ± 0.05	1.11 ± 0.06	
	1.10–1.25; 5	1.18–1.33; 6	1.07–1.21; 16	1.02–1.19; 25	1.03–1.16; 11	1.08–1.23; 7	1.16; 1.22; 2	1.13–1.27; 6	1.04–1.19; 5	
**TL/HBL**	86.46%	76.37%	91.00%	91.51%	91.28%	98.78%	93.33%	87.68%	86.12%	83.50%
**GNB/PL**	51.80%	55.74%	53.94%	54.24%	52.40%	54.43%	53.45%	52.87%	53.96%	53.61%

**Table 3.** Character loadings, eigenvalues, and percent variance explained on the first two components of a principal components analysis and the five canonical axes discriminant function analyses of the genus *Uropsilus*.

Variables	PCA		DA				
	1	2	1	2	3	4	5
LUTR	0.832	0.313	0.243	−0.079	0.275	−0.457	−1.450
LBTR	0.807	0.256	0.353	−0.398	−0.934	0.619	0.266
PL	0.781	0.283	1.259	−0.271	−0.119	−0.180	0.735
Id-Gol	0.770	0.276	0.952	−0.544	0.215	−0.133	−0.329
ML	0.667	0.302	0.625	1.142	−0.602	−0.854	−0.317
MPL	0.647	0.198	−0.754	−0.556	0.599	1.207	0.877
P^4^-M^3^	0.625	0.549	−0.374	0.322	−0.680	−0.106	−0.944
M^1^-M^3^	0.604	0.598	0.003	0.058	0.666	0.184	0.848
APB	0.592	0.228	−0.092	−0.052	−0.302	0.220	0.267
GBUM	0.138	0.892	0.264	0.459	−0.083	0.641	0.649
GBSn	0.211	0.854	−0.276	0.421	0.475	−1.268	0.024
GBLM	0.288	0.741	−0.079	−0.062	0.249	0.249	0.341
BBP^1^-P^2^	0.368	0.698	0.591	0.459	−0.474	0.373	−0.416
PPB	0.252	0.603	−0.339	−0.393	0.229	0.047	0.356
Id-Coh	0.321	0.067	−1.660	0.156	0.770	0.207	−0.678
HVR	0.354	0.402	−0.601	0.166	−0.025	0.164	−0.487
Coh-M_3_	0.484	−0.006	−0.291	−0.622	0.580	0.164	1.254
GNB	0.111	0.524	0.759	0.270	0.023	−0.253	−0.493
HB	0.293	0.112	0.047	−0.434	0.608	−0.251	−0.004
BS	0.179	0.218	0.195	0.256	−0.424	0.515	0.104
LBO	0.184	−0.070	−0.203	−0.254	−0.585	−0.497	0.476
Eigenvalues	12.020	2.211	8.426	2.829	0.973	0.563	0.263
Percent variance explained (%)	57.24%	10.53%	64.70%	21.70%	7.20%	4.30%	2.00%

Both the molecular and morphological analyses indicate that *Uropsilus* sp. nov. is diagnosable from all other recognized species of the genus *Uropsilus*. Based on the diagnosis and monophyly-based phylogenetic species concept (Mayden 1997; Gutierrez and Garbino 2018), we recognize it as a new species, which we formally describe below.

### ﻿Taxonomic account

#### ﻿ Uropsilus huanggangensis

Taxon classificationAnimaliaEulipotyphlaTalpidae

Chen, Jiang & Ren sp. nov.

C1A76326-3EEF-50FF-8550-CE405577F1E1

https://zoobank.org/160BAE4A-EBEB-4177-8A50-ADF35E932D4C

Figs 4 , 5

##### Suggested common name.

Huanggang shrew mole; Chinese common name: 黄岗鼩鼹.

##### Type materials.

***Holotype***: AHNU 2022013, an adult male collected by Zhongzhen Chen in June 2022 from Mount Huanggang, Wuyishan National Park, Jiangxi Province, China (27°58'53"N, 117°47'2.4"E, altitude 2061 m a.s.l.). The dried skin and cleaned skull are deposited in ANHU. ***Paratypes***: AHNU 2022014, AHNU 2022053, AHNU 2022054, and AHNU 2022055; 4 adult specimens collected from Mount Huanggang, Wuyishan National Park, Jiangxi Province, China at elevations between 1830 and 2060 m a.s.l. The specimens are deposited in AHNU.

##### Etymology.

The specific name *huanggangensis* is derived from Mount Huanggang, the type locality of the new species; the Latin adjectival suffix -*ensis* means “belonging to”.

##### Diagnosis.

The dorsal pelage of *U.huanggangensis* is dark chocolate-brown. The snout is the longest of any species in the genus. The first incisor I^1^ is wide and shows an enlargement at the apex of the rostrum, with a visible gap to I^2^. C_1_ is larger than P_1_, and P_1_ and P_3_ are similar in size. Tail is slim and relatively short, averaging 86% of head and body length. The tufts at the tail tip are short. The lacrimal foramen and infraorbital foramen are similar in size. The coronoid process is pointed and converges more upward with an incisive tip. The dental formula is I 2/1, C1/1, P 4/4, M 3/3 = 38.

##### Description.

*Uropsilushuanggangensis* is a medium-sized species of *Uropsilus* (HBL = 72 ± 1 mm, PL = 21.44 ± 0.47 mm; Table 2). The dorsal pelage is dark chocolate-brown, consisting of brown fur with a light grey base; the ventral fur is slightly paler. The snout is very long, at about 12 mm, and is the longest in the genus. The tail is slim and relatively short (TL = 63 ± 3 mm, 57–65 mm), about 86% of the combined head and body length. The tail is black above and slightly paler below, with a sparse tuft of short hair at its tip. The hind foot is covered with short black hair; its length is 13–14 mm and constitutes approximately 18% of the combined head and body length.

The outlines of the skull are rounded, and there is a complete zygomatic arch. The rostrum is relatively long, the braincase is narrow, and the proportion of GNB and PL is 51.8%, which is the smallest of any species in the genus (GNB/PL > 52.4% in other species). The zygomatic arches are stout and only slightly bow outward. The lacrimal foramen and infraorbital foramen are similar in size.

The dental formula is I 2/1, C1/1, P 4/4, M 3/3 = 38. I^1^ is large and wide, causing the enlargement at the apex of the rostrum. I^1^ is bigger than I^2^, and there is a visible gap between them. C^1^ is almost equal to P^1^, while P^3^ is smaller. P^2^ is larger than P^1^ and P^3^. The first upper molar M^1^ and second upper molar M^2^ are large, and have well-developed, W-shaped lateral cusps. In contrast, the third upper molars M^3^ are reduced.

The body of the mandible is long and slender. The coronoid process is high, pointed, and curved to the posterior, with an incisive tip pointing straight to the posterior, resembling the outline of a sickle. The angular process is long, rounded, and points downward at roughly 45°. The first lower premolar (P_1_) is slightly smaller than the lower canine C_1_. P_1_ and P_3_ are similar in size. M_2_ is W-shaped and larger than M_1_ and M_3_. M_3_ is slightly smaller than M_1_ (Fig. 6).

##### Comparison.

Among other *Uropsilus* species, *U.huanggangensis* is morphologically most similar to *U.dabieshanensis* and *U.gracilis*. However, the new species can be distinguished from them by many characteristics.

**Figure 5.**
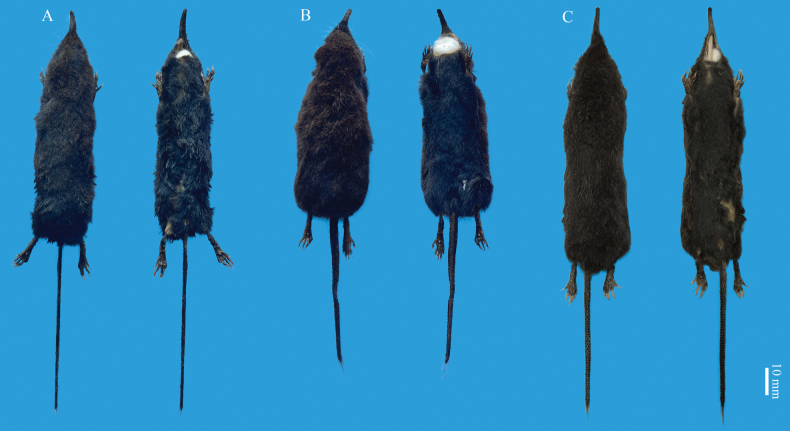
Dorsal and ventral views of three *Uropsilus* species **A***U.huanggangensis* sp. nov. **B***U.gracilis***C***U.dabieshanensis*.

**Figure 6.**
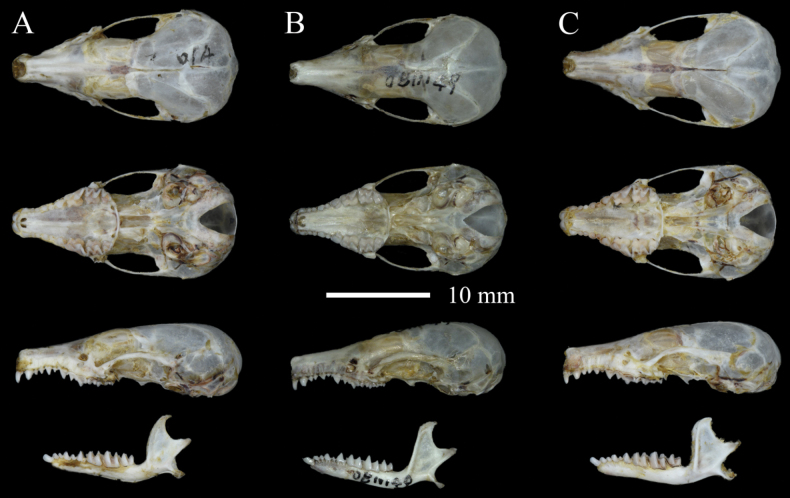
Dorsal, ventral, and lateral views of the skull and lateral views of the mandible of three *Uropsilus* species **A***U.huanggangensis* sp. nov. **B***U.gracilis***C***U.dabieshanensis*.

Compared to *U.dabieshanensis*, *U.huanggangensis* has darker fur, a relatively longer and slimmer tail, and a much larger hindfoot and ear, despite that the heads and body lengths of the two species are almost the same (Table 2; Fig. 5). The tail of *U.huanggangensis* (TL = 63 ± 3 mm) is relatively longer than *U.dabieshanensis* (TL = 56 ± 3 mm). Most individuals of *U.huanggangensis* (4 of 5) have a tail length of more than 63 mm, while most individuals of *U.dabieshanensis* (5 of 6) have a tail length less than 57 mm. The hairs on the tail (bristle hairs) of *U.huanggangensis* are shorter and sparser than those in *U.dabieshanensis*, and the tufts at the tail tip of *U.dabieshanensis* appear much longer. The skull of *U.huanggangensis* is much slenderer than in *U.dabieshanensis* (Fig. 6), and the proportion of GNB and PL (CB / GLS = 53.96%) in *U.dabieshanensis* is greater than that in *U.huanggangensis* (GNB / PL = 51.80%). The coronoid process is pointed and curved to the posterior in *U.huanggangensis*, while the coronoid process of *U.dabieshanensis* is high and straight, with a squared tip.

Compared to *U.gracilis*, the dorsal pelage of *U.huanggangensis* is much darker. The snout of *U.huanggangensis* is longer, and the incisor is larger than that of *U.gracilis*. The tail of *U.huanggangensis* (TL/HBL = 86%) is relatively shorter than *U.gracilis* (TL / HBL = 91%) in proportion, and the tufts at the tail tip of *U.huanggangensis* are much shorter than those in *U.gracilis*. In terms of body size, *U.huanggangensis* is relatively larger than *U.gracilis* for most external and craniomandibular measurements (Table 2). In particular, the range of Id-Gol (*U.huanggangensis* 12.90–13.03 mm vs *U.gracilis* 11.46–12.68 mm) between the two species does not overlap. The coronoid process of *U.gracilis* is high and squared, similar to that of *U.dabieshanensis*, but differs from that of *U.huanggangensis*.

Compared to *U.atronates* and *U.nivatus*, the dark chocolate-brown dorsal pelage of *U.huanggangensis* differs from the chestnut red of *U.atronates* and the black-gold pellage of *U.nivatus*. Meanwhile, *U.huanggangensis* is larger than both *U.atronates* and *U.nivatus* for most external and craniomandibular measurements (Table 2).

The pelage color of *U.huanggangensis* is dark chocolate-brown, which is much lighter than the black pelage of *U.investigator*. The ears of *U.huanggangensis* are relatively larger (EL = 9.20 ± 0.45 mm, range 9.00–10.00 mm) than that of the *U.investigator* (EL = 7.95 ± 0.98 mm, range 6.50–9.50 mm). The color of *U.huanggangensis* is uniform compared to the bicolored tail of *U.investigator*. Also, the P_1_ of *U.investigator* is larger than P_3_, while P^1^ and P_3_ of *U.huanggangensis* are similar in size.

Compared to the upward orbital process of *U.fansipanensis*, the orbital process of *U.huanggangensis* is downward. The lacrimal foramen of *U.fansipanensis* is larger than infraorbital foramen, while the two are of similar size in *U.huanggangensis*.

The dental formula of *U.huanggangensis* is I 2/1, C1/1, P 4/4, M 3/3 = 38, which can be easily distinguished from *U.soricipes* (dental formula I 2/1, C 1/1, P 3/3, M 3/3 = 34), *U.andersoni* (dental formula I 2/2, C1/1, P 4/3, M 3/3 = 38), and *U.aequodonenia* (dental formula I 2/2, C 1/1, P 3/3, M 3/3 = 36).

##### Distribution and ecology.

*Uropsilushuanggangensis* is currently known only from the type locality on Mount Huanggang, Wuyishan National Park, Jiangxi Province, eastern China, where pecimens were collected at elevations between 1830 and 2060 m a.s.l. Coniferous forests and shrub meadows, with abundant rocks on the ground, dominate the habitat in this area.

## ﻿Discussion

For a long time, it was believed that the genus *Uropsilus* was only distributed in the mountains of southwestern China and adjacent Myanmar (Wan et al. 2013; Kryštufek and Motokawa 2018). Hu et al. (2021b) expanded the known distribution of the genus by reporting the presence of *U.dabieshanensis* on Dabie Mountain, Anhui, eastern China, which represents the easternmost distribution of genus. In the present study, through integrating morphological and molecular approaches, we demonstrate that the isolated population on Mount Huanggang is distinct from all nominal species of *Uropsilus* and recognize it as a new species, *U.huanggangensis*.

In line with previous studies (Wan et al. 2013; Hu et al. 2021b), our phylogenetic analyses reveal that *Uropsilus* species can be sorted into two distinct lineages. One lineage includes *U.investigator* and *U.* sp. 1, occupying the basal position of the genus, and the other lineage exhibits a widespread distribution throughout China. Although *U.huanggangensis* consistently forms a monophyletic group with strong support in all phylogenetic trees, it is worth noting that the phylogenetic trees of *Uropsilus* species display considerable instability, as previously reported in the literature. Additionally, eight putative new species (*Uropsilus* sp. 1–8) have been identified but not yet officially described; there is a high level of cryptic diversity and extensive cryptic diversification within the genus. Broader sampling, in-depth gene sequencing, and morphological analysis are needed to improve the understanding of the genus.

As the easternmost occurring species of *Uropsilus*, our discovery of *U.huanggangensis* significantly expands our knowledge of the geographic distribution of the genus and contributes to our understanding of its macroevolution. The divergence of *U.huanggangensis* is estimated in the early Pleistocene (1.98 Ma, 95% CI = 0.88–2.99). Global cooling and drying events during this period (Qiu and Li 2005; Ge et al. 2013), as well as the isolation effects of Wuyi Mountain and Yangtze River, may have been critical in the divergence of *U.huanggangensis*, and Mount Huanggang may have provided a refuge for *U.huanggangensis* during the ice age. Recently, several new small mammal species have been described in eastern China, such as *Chodsigoadabieshanensis*Chen et al., 2022, *Crociduadongyangjiangensis*Liu et al., 2020, and *Typhlomyshuangshanensis* Hu et al., 2021, indicating that biodiversity in the region is severely underexplored (Hu et al. 2021a; Chen et al. 2022). The description of *U.huanggangensis* in the Wuyi Mountains region highlights the overlooked biodiversity of the mountains of eastern China. It is therefore crucial to conduct further comprehensive investigations and taxonomic studies on small mammals in this region to gain a deeper understanding of the biodiversity of this region.

## Supplementary Material

XML Treatment for Uropsilus huanggangensis

## ﻿Additional information

### Author ORCIDs

Xueyang Ren https://orcid.org/0000-0002-3137-0309

Yifan Xu https://orcid.org/0000-0002-8433-1768

Hongfeng Yao https://orcid.org/0009-0007-2493-2135

Laxman Khanal https://orcid.org/0000-0003-2411-3627

Xuelong Jiang https://orcid.org/0000-0003-2052-2490

Zhongzheng Chen https://orcid.org/0000-0003-3821-0145

### Data availability

All of the data that support the findings of this study are available in the main text or Supplementary Information.

## ﻿Appendix 1

Specimens examined for this study. Abbreviations: KIZ, Kunming Institute of Zoology, Chinese Academy of Sciences; AHNU, Anhui Normal University.

*Uropsilushuanggangensis* sp. nov. (*n* = 5): Mount Huanggang, Jiangxi (AHNU 2022013-014, AHNU 2022053-055).

*U.aequodonenia* (*n* = 2): Mount Daliangshan, Yuexi, Sichuan (KIZ 0906075, KIZ SC2110638).

*U.andersoni* (*n* = 6): Mount Erlang, Tianquan, Sichuan (KIZ SC2110004, KIZ SC2110014-015, KIZ Z201505738-739, KIZ Z201505755).

*U.atronates* (*n* = 25): Mount Laobieshan, Yongde, Yunnan (KIZ 0212145, KIZ 0212185, KIZ 0212187, KIZ 0212190, KIZ 0212309, KIZ 0212314, KIZ 0212332, KIZ 0212334, KIZ 0212339, KIZ 0212381-382, KIZ 0212413); Caojian, Yunlong, Yunnan (KIZ 0904362, KIZ 0904420-403); Laowo, Lushui, Yunnan (KIZ 2012121120); Wayao, Baoshan, Yunnan (KIZ H2004, KIZ H2007, KIZ H2013-14, KIZ H2040, KIZ H2052, KIZ H2054, KIZ H2073, KIZ H2090).

*U.dabieshanensis* (*n* = 6): Dabie Mountain, Anhui (AHNU 202109002, AHNU 202109115, AHNU 202109302, AHNU 202109337, AHNU 202109409, AHNU 202109457).

*U.gracilis* (*n* = 16): Mount Jinfoshan, Nanchuan, Chongqing (KIZ Y204002, KIZ Y204043); Mount Jiaozishan, Dongchuan, Yunnan (KIZ 0810003, KIZ 0810137, KIZ0810159-160, KIZ 0810191-192, KIZ 0810245, KIZ 0810274, KIZ 0810488, LIZ 0810504); Mount Jiaozishan, Luquan, Yunnan (KIZ 0811149, KIZ 0811175-176); Mount Wumeng, Zhaotong, Yunnan (KIZ 201309117).

*U.investigator* (*n* = 11): Mount Gaoligong, Gongshan, Yunnan (KIZ PM1311422, KIZ PM1312467, KIZ PM1312511, KIZ PM1312570, KIZ PM1312600, KIZ 201211136, KIZ 201211160, KIZ 201211575, KIZ GLGS1945).

*U.nivatus* (*n* = 7): Mount Diancang, Dali, Yunnan (KIZ DL1110269); Mount Cangshan, Dali, Yunnan (KIZ H0122); Mount Yunling, Deqin, Yunnan (KIZ DQ1204010, KIZ DQ1204049); Mount Yunling, Weixi, Yunnan (KIZ WX1204157, KIZ WX1204284); Mount Wushan, Changyang, Hubei (KIZ CY2010353).

*U.soricipes* (*n* = 5): Beichuan, Sichuan (KIZ 0905021); Shawan, Leshan, Sichuan (KIZ 0905283, KIZ 0905292, KIZ 0905308); Mount Daxiangling, Shimian, Sichuan (KIZ 0905409).

## Supplementary materials

Supplementary material 1 PCR amplification primer sequences for *CYT B*, *12S*, *RAG1*, *RAG2*, and *PLCB4* Data type xlsx https://binary.pensoft.net/file/949003This dataset is made available under the Open Database License (http://opendatacommons.org/licenses/odbl/1.0/). The Open Database License (ODbL) is a license agreement intended to allow users to freely share, modify, and use this Dataset while maintaining this same freedom for others, provided that the original source and author(s) are credited.Xueyang Ren, Yifan Xu, Yixian Li, Hongfeng Yao, Yi Fang, Laxman Khanal, Lin Cheng, Wei Zeng, Xuelong Jiang, Zhongzheng Chen

Supplementary material 2 Samples and sequences used for molecular analyses Data type xlsx Explanation note New sequences generated in this study are shown in bold. https://binary.pensoft.net/file/949004This dataset is made available under the Open Database License (http://opendatacommons.org/licenses/odbl/1.0/). The Open Database License (ODbL) is a license agreement intended to allow users to freely share, modify, and use this Dataset while maintaining this same freedom for others, provided that the original source and author(s) are credited.Xueyang Ren, Yifan Xu, Yixian Li, Hongfeng Yao, Yi Fang, Laxman Khanal, Lin Cheng, Wei Zeng, Xuelong Jiang, Zhongzheng Chen

Supplementary material 3 Partitioning schemes and molecular evolution model used in mitochondrial–nuclear concatenated gene tree estimations Data type xlsx https://binary.pensoft.net/file/949005This dataset is made available under the Open Database License (http://opendatacommons.org/licenses/odbl/1.0/). The Open Database License (ODbL) is a license agreement intended to allow users to freely share, modify, and use this Dataset while maintaining this same freedom for others, provided that the original source and author(s) are credited.Xueyang Ren, Yifan Xu, Yixian Li, Hongfeng Yao, Yi Fang, Laxman Khanal, Lin Cheng, Wei Zeng, Xuelong Jiang, Zhongzheng Chen

Supplementary material 4 Independent-sample *t*-test Data type xlsx Explanation note Independent-sample t-test of the variances of measurements highly correlated with PC1 and PC2 between *Uropsilus* sp. nov. and *U.atronates*, *U.dabieshanensis*, *U.gracilis*, *U.investigator*, and *U.nivatus*. https://binary.pensoft.net/file/949006This dataset is made available under the Open Database License (http://opendatacommons.org/licenses/odbl/1.0/). The Open Database License (ODbL) is a license agreement intended to allow users to freely share, modify, and use this Dataset while maintaining this same freedom for others, provided that the original source and author(s) are credited.Xueyang Ren, Yifan Xu, Yixian Li, Hongfeng Yao, Yi Fang, Laxman Khanal, Lin Cheng, Wei Zeng, Xuelong Jiang, Zhongzheng Chen
